# Characterization of exopolysaccharide-producing lactic acid bacteria from Taiwanese ropy fermented milk and their application in low-fat fermented milk

**DOI:** 10.5713/ab.21.0251

**Published:** 2021-08-25

**Authors:** Ker-Sin Ng, Yu-Chun Chang, Yen-Po Chen, Ya-Hsuan Lo, Sheng-Yao Wang, Ming-Ju Chen

**Affiliations:** 1 Department of Animal Science and Technology, College of Bioresources and Agriculture, National Taiwan University, Taipei 10617, Taiwan; 2 Department of Animal Science, National Chung Hsing University, Taichung 40227, Taiwan; 3 The iEGG and Animal Biotechnology Center, National Chung Hsing University, Taichung 40227, Taiwan

**Keywords:** Exopolysaccharides, Lactic Acid Bacteria, Low-fat Fermented Milk, Taiwanese Ropy Fermented Milk

## Abstract

**Objective:**

The aim of this study was to characterize the exopolysaccharides (EPS)-producing lactic acid bacteria from Taiwanese ropy fermented milk (TRFM) for developing a clean label low-fat fermented milk.

**Methods:**

Potential isolates from TRFM were selected based on the Gram staining test and observation of turbid suspension in the culture broth. Random amplified polymorphic DNA-polymerase chain reaction, 16S rRNA gene sequencing, and API CHL 50 test were used for strain identification. After evaluation of EPS concentration, target strains were introduced to low-fat milk fermentation for 24 h. Fermentation characters were checked: pH value, acidity, viable count, syneresis, and viscosity. Sensory evaluation of fermented products was carried out by 30 volunteers, while the storage test was performed for 21 days at 4°C.

**Results:**

Two EPS-producing strains (APL15 and APL16) were isolated from TRFM and identified as *Lactococcus* (*Lc.*) *lactis* subsp. *cremoris*. Their EPS concentrations in glucose and lactose media were higher than other published strains of *Lc. lactis* subsp. *cremoris*. Low-fat fermented milk separately prepared with APL15 and APL16 reached pH 4.3 and acidity 0.8% with a viable count of 9 log colony-forming units/mL. The physical properties of both products were superior to the control yogurt, showing significant improvements in syneresis and viscosity (p<0.05). Our low-fat products had appropriate sensory scores in appearance and texture according to sensory evaluation. Although decreasing viable cells of strains during the 21-day storage test, low-fat fermented milk made by APL15 exhibited stable physicochemical properties, including pH value, acidity, syneresis and sufficient viable cells throughout the storage period.

**Conclusion:**

This study demonstrated that *Lc. lactis* subsp. *cremoris* APL15 isolated from TRFM had good fermentation abilities to produce low-fat fermented milk. These data indicate that EPS-producing lactic acid bacteria have great potential to act as natural food stabilizers for low-fat fermented milk.

## INTRODUCTION

The global market of low-fat dairy products, including yogurt, milk, cheese, and ice-cream, has been expanding considerably since the early 20th century [1]. Low-fat products are often served to restrict the diet of patients with metabolic disorders, such as obesity, diabetes, and cardiovascular diseases, however it is now common for consumers to incorporate such products in daily meals due to the health concerns that come with excessive intake of fat. Several studies indicate that low-fat dairy consumption could reduce risk factors of human metabolic syndrome [2] when comparing with whole-fat dairy products.

Despite the increasing need for low-fat foods, reduction of fat in these products often lead to inferior texture, odor, and taste. Fat is a precursor of aromatic volatiles as the oxidation of lipids derives an adequate amount of ketones, lactones, and others for flavor development of dairy foods [3]. In contrast to protein and carbohydrate, fat is an important solvent to retain hydrophobic compounds in foods. Brauss et al [4] found that fat percentage in yogurt lower than 3.5% was not sufficient to dissolve lipophilic volatiles and exhibited larger particle size, which affected favor release and lowered viscosity in the final product.

Exopolysaccharides (EPS) naturally secreted by microorganisms have been identified as prospective fat replacers to modify the textural and rheological properties of food matrixes. EPS exist in long chains of homo- or heteropolysaccharides as repeating units of glucose, fructose, galactose, rhamnose, and others. These molecules can be natural water-binding agents that improve moisture retention and reduce wheying off in dairy products. *Bifidobacterium (B.) bifidum*, *B. breve*, *B. longum* subsp. *infantis*, *Lactobacillus (Lb.) fermentum*, and *Lb. mucosae* have been reported as EPS producers that contributed to the viscosity of low-fat yogurts [5–7] through their interactions with milk protein networks [8].

Viili, a traditional Finnish fermented milk, is one example of a commercialized product with strong ropy properties, and with great extension ability as a result of EPS. However, there is no report on the slime-forming strains from viili, which are good sources of EPS, in the application of low-fat fermented milk. Taiwanese ropy fermented milk (TRFM) is a viscous beverage similar to viili [9]. Thus, EPS-producing strains from villi (or TRFM) could be an effective solution to improve the texture of low-fat fermented milk, and simultaneously confer health benefits. In the present study, EPS-producing isolates from TRFM were identified using molecular methods and carbohydrate utilizing test. After evaluation of EPS concentration, we tested the fermentation characters of target strains in making of low-fat fermented milk when compared to commercial yogurt, and also examined the condition of the storage test for 21 days. The final aim of this study was to develop an innovative low-fat fermented milk with suitable perception without the use of food additives.

## MATERIALS AND METHODS

### Screening of exopolysaccharides-producing strains from Taiwanese ropy fermented milk

The TRFM was prepared in our lab by repeated batch fermentation (16 to 18 h at 20°C) using pasteurized fresh cow milk. After serial dilutions, TRFM was plated on de Man, Rogosa, and Sharpe (MRS) agar (Acumedia Manufacture, Lansing, MI, USA) and incubated aerobically at 26°C for 24 h. Isolated strains were cultured in MRS broth at 26°C for 24 h and vortexed for 3 s to observe their cell pellets, those remained intact at the bottom of culture tubes with a turbid suspension were regarded as potential EPS producers [10]. The isolates were classified by Gram-staining with BaSO Rapid Gram Stain (BASO BIOTECH CO., LTD., New Taipei, Taiwan).

### DNA extraction

For Gram-positive EPS-producing bacteria, DNA was extracted as described by Watanabe et al [11] with modifications. One milliliter of bacterial culture was centrifuged at 12,000×*g*, 4°C for 5 min. The cell pellet was suspended in 500 μL of DNA extraction buffer (A: 200 m*M* Tris-HCl, 80 m*M* ethylenediaminetetraacetic acid (EDTA), pH 9.0, B: 10% sodium dodecyl sulfate; A:B = 5:1), vortexed with 0.3 g of glass beads (0.1 mm in diameter) at 3,000 rpm (Digital Vortex-Genie 2, Scientific Industries Inc., Bohemia, New York, USA) for 10 min, and centrifuged at 12,000×*g* and 4°C for 5 min. Four hundred microliters of the supernatant were mixed with 400 μL of phenol-chloroform-isoamyl alcohol solution (25:24:1 saturated with 10 mM Tris, pH 8.0, and 1 mM EDTA) and centrifuged at 12,000×*g* and 4°C for 5 min. The collected supernatant (250 μL) was mixed with 25 μL of 3 M sodium acetate solution and 250 μL of isopropanol. After centrifugation, the precipitate was washed with 500 μL of 70% ethanol, and the supernatant was removed. The precipitate was then washed several times with absolute ethanol. Finally, the pelleted DNA was dried overnight under a hood and stored in 200 μL of TE buffer (10 m*M* Tris-HCl, 1 m*M* EDTA, pH 8.0) at −20°C for further experiments.

### Random amplified polymorphic DNA-polymerase chain reaction typing

Random amplified polymorphic DNA (RAPD)-polymerase chain reaction (PCR) fingerprinting was performed to differentiate the potential isolates into groups. A 25 μL mixture containing 2 m*M* 10× *Ex Taq* buffer (Takara Bio Inc., Shiga, Japan), 1 m*M* MgCl_2_, 200 μ*M* dNTP, 1 U *Ex Taq* DNA polymerase, 0.16 μ*M* RAPD primer (p1281, 5′-AACGCGCAAC-3′) [11], and 10 ng of template DNA was amplified with a program composed of 1 cycle of 94°C for 2 min; 6 cycles of 94°C for 30 s, 36°C for 60 s, and 72°C for 90 s; 30 cycles of 94°C for 20 s, 36°C for 30 s, and 72°C for 90 s; and finally, 1 cycle of 72°C for 3 min (Biometra T3000 thermocycler; Analytik Jena, Göttingen, Germany) [11]. PCR products were electrophoresed at 50 V for 1 h on a 1.5% (w/v) agarose gel.

### Strains identification

#### 16S rRNA gene sequencing

Primers 8F (5′-AGAGTTTGAT CMTGGCTCAG-3′) and 15R (5′-AAGGAGGTGATCCA RCCGCA-3′) were used to amplify the fragments of 16S rRNA gene [11]. The amplification program was composed of 1 cycle of 94°C for 2 min; 30 cycles of 94°C for 30 s, 58°C for 30 s, and 72°C for 90 s; and finally, 1 cycle of 72°C for 10 min. Sequencing was conducted with ABI 3730 XL DNA analyzer (Applied Biosystems, Foster City, CA, USA) by Genomics Bio Sci & Tech Co. Ltd. (New Taipei, Taiwan), and the obtained data were aligned and assembled with Chromas v2.23 (Technelysium Pty. Ltd., Queensland, Australia), GENETYX v5.1 (Software Development Co., Tokyo, Japan), and GENETYX ATSQ v1.03 (Software Development Co., Japan). To determine the 16S rRNA gene sequence similarities, the nucleotide BLAST program in National Center for Biotechnology Information (https://blast.ncbi.nlm.nih.gov/Blast.cgi) was accessed. A phylogenetic tree was constructed by the neighbor-joining method with Kimura’s two-parameter model, and *Escherichia coli* ATCC 11775^T^ was used as an outgroup. Tree topology was evaluated with 1,000 trials of bootstrap value using MEGA7 v7.0.14 [12].

#### Carbohydrate fermentation

The carbohydrate utilizing ability of selected lactic acid bacteria (LAB) was evaluated with API 50 CHL (bioMérieux, Inc., Marcy l’Etoile, France). Briefly, the bacterial suspension was added to each tube of the strip (incubated at 30°C), and color change was recorded after 24 h and 48 h. Each tube contained a certain carbohydrate and bromocresol purple as an indicator, and the color change from purple to yellow was considered as a positive reaction. This assay was examined with a protocol provided by the manufacturing company, in which 49 carbohydrates were tested, and 52 species of LAB were included in the database for comparison.

### Exopolysaccharides assay of culture broth

To evaluate the effect of carbon sources on EPS production, Lactobacilli MRS broth without dextrose (Alpha Biosciences Inc., Baltimore, MD, USA) was supplemented with 2% glucose or 2% lactose for bacterial cultivation at 26°C for 24 h. The EPS assay was performed as described previously [13]. The bacterial culture was mixed with 4% (v/v) of trichloroacetic acid (Sigma-Aldrich Co., St. Louis, MO, USA) and left overnight at room temperature; then, it was centrifuged at 2,330×g for 1 h to collect the supernatant. An equal volume of absolute ethanol was added and left overnight to precipitate EPS (8,000×g, 5 min), and the supernatant was discarded. After three times of ethanol precipitation, the EPS pellet was dried at 60°C for 30 min, and finally dissolved in 1 mL double distilled water. The concentration of EPS was measured by a phenol-sulfuric method with absorbance at 450 nm. D(+)-glucose was used in the establishment of the standard curve.

### Production of low-fat fermented milk and storage test

For activation of EPS-producing strains, APL15 and APL16 were precultured twice in MRS broth with 1% (v/v) inoculum at 26°C for 24 h, then the bacterial cultures were centrifuged (1,770×g, 10 min at 4°C) and resuspended in 10 mL of 0.85% saline solution, for two times. To prepare low-fat fermented milk, these were inoculated at 1% (v/v) into fresh, low-fat milk (fat content was 14 g/L; Wei Chuan Foods Corporation, Taipei, Taiwan) individually, and incubated at 26°C for 24 h. For preparation of the control yogurt, the thermophilic yogurt culture YC-380 (Chr. Hansen Holding A/S, Hoersholm, Denmark) consisting of *Streptococcus (S.) thermophilus* and *Lb. delbrueckii* subsp. *bulgaricus* was inoculated into pasteurized fresh low-fat milk at 40°C for 8 h. After fermentation, the samples were packed and stored at 4°C for further analysis. The physicochemical and microbial properties of the samples were evaluated every 7 days during the 21 days of storage.

#### Physicochemical characteristics

The pH of fermented milk was measured with a Lab 850 pH meter (SI Analytics GmbH, Berlin, Germany) while its titratable acidity was assessed according to ISO 6901:2010 [14]. Syneresis was determined as described by Mani-Lopez et al [15]. With regard to the viscosity of fermented milk, RST-CPS Touch Rheometer (Brookfield Engineering Laboratories Inc., Middleboro, MA, USA) was used to examine it with a spindle of RPT-50 (using parallel geometry at 1 mm gap), and the shear rate was kept as 30 s^−1^ at 7°C [16].

#### Microbial analysis

One milliliter of fermented milk was diluted with 9 mL of sterile 0.85% saline and mixed thoroughly. Serial 10-fold dilutions were performed, and 0.1 mL aliquots of the appropriate dilutions were directly inoculated onto MRS agar. After incubation at 26°C for 2 d, the colonies were counted and expressed as colony-forming units per milliliter (CFU/mL).

#### EPS assay of fermented milk

This assay was adjusted as described by Rimada and Abraham [17]. Fermented milk was refrigerated at 4°C overnight. After boiling for 30 min and centrifuging at 10,000×g, 20°C for 30 min, the collected supernatant was dialyzed (molecular weight cut-off for the dialysis membranes: 3,500; Membrane Filtration Products Inc., Seguin, TX, USA) for 48 h at 4°C against double distilled water. Then, EPS concentration was examined using a modified phenol-sulfuric method. Absorbance at 485 nm was recorded with D(+)-glucose as a standard.

### Sensory evaluation

Thirty volunteers were recruited for the consumer acceptance test of low-fat fermented milk prepared by strains APL15 and APL16, and control low-fat yogurt (fat content was 15 g/L, supplemented with skim milk powder and citrus pectin; Standard Foods Co., Ltd., Taoyuan, Taiwan). They were requested to provide responses on the samples in terms of appearance, aroma, texture, flavor, and overall evaluation with the 9-level hedonic test (1, extremely bad; 2, very bad; 3, bad; 4, moderately bad; 5, fair; 6, moderately good; 7, good; 8, very good; 9, extremely good) [18].

### Statistical analysis

Sensory evaluation was accessed with non-parametric statistics, and the other experiments were carried out with three replicates and analyzed using the analysis of variance general linear model procedure in Statistical Analysis Systems (SAS) software. Comparisons between two groups and multiple groups were conducted with unpaired Student *t*-test and Tukey’s test, respectively.

## RESULTS AND DISCUSSION

### Exopolysaccharides-producing strains from Taiwanese ropy fermented milk

#### Strains identification

**Figure 1:**
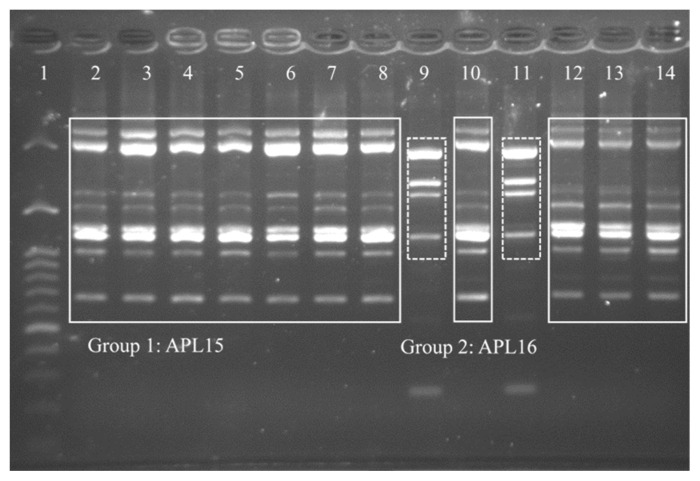
Random amplified polymorphic DNA-polymerase chain reaction (RAPD-PCR) profiles of 13 selected isolates from Taiwanese ropy fermented milk (TRFM) after amplification with RAPD-B primer. Lane 1, 100 bp molecular weight DNA ladder; lanes 2–8, 10, and 12–14, group 1 indicates APL15; lanes 9 and 11, group 2 indicates APL16.

**Figure 2:**
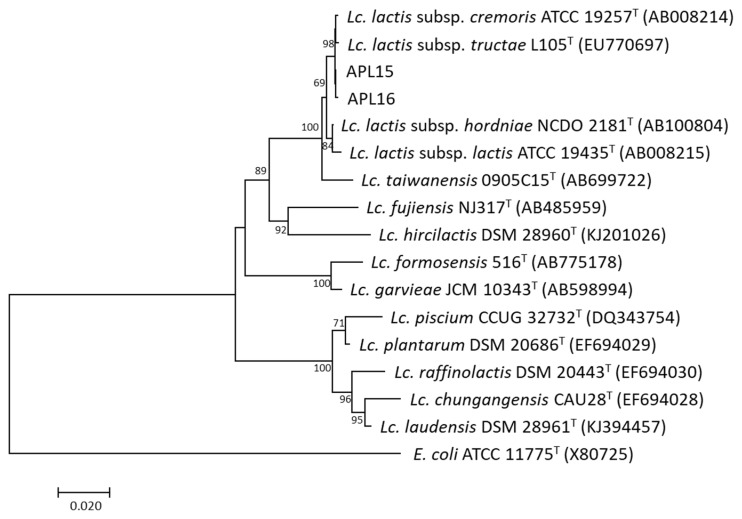
Phylogenetic relationships of the strains APL15 and APL16 to their related species in the genus *Lactococcus* based on 16S rRNA gene sequences. *Escherichia coli* ATCC 11775^T^ was used as an outgroup. Bootstrap values with 1,000 replications are given at nodes. The bar shows 2% of sequence divergence.

The TRFM is a domestic fermented beverage with high viscosity as a product of microbial fermentation. To identify potential EPS-producing bacteria, 40 isolates were isolated from TRFM and purified, of which 13 Gram-positive isolates showing ropy properties were cultured for DNA extraction. By RAPD-PCR fingerprinting, 13 EPS-producing isolates from TRFM were divided into two distinct groups, Group 1 (comprised 11 isolates) and Group 2 (comprised two isolates). This result demonstrated that 11 isolates in Group 1 and two isolates in Group 2 were clones of a single strain, respectively. Therefore, one isolate from each group was chosen, and assigned the strain names as APL15 and APL16 for further analyses (Figure 1). 16S rRNA gene sequences similarities between strains APL15 and APL16, and their closest taxa, *Lc. lactis* subsp. *cremoris* and *Lc. lactis* subsp. *tructae* were greater than 99% (Table 1). A phylogenetic tree was constructed of target strains with their closely related species in the genus *Lactococcus* (Figure 2). APL15 and APL16 were clustered with *Lc. lactis* subsp. *cremoris* and *Lc. lactis* subsp. *tructae* with 98% of reproducibility among 1,000 bootstrap trees. Tanigawa et al [19] reported that *Lc. lactis* subsp. *cremoris* strains were clearly differentiated from Lc. lactis subsp. lactis based on ribosomal subunits (30S/S20, 50S/L31, L35), *recA* gene and 16S rRNA gene (especially in V1 region) sequences, and ribose fermentation. By analyzing the V1 region of 16S rRNA gene, we found that APL15 and APL16 were not identified as *Lc. lactis* subsp. *lactis* (Supplementary Figure S1).

Phenotypic features can also be used to define the subspecies of *Lc. lactis* isolates, such as growing condition, sugar fermentation, and enzymatic activity. For further identification, carbohydrate fermentation reactions of APL15 and APL16 were identified with API LAB database. Both strains had identical fermentation profiles, and were identified as *Lc. lactis* subsp. *cremoris* with 98.7% identity. In addition, based on the utilization of ribose, sucrose and *β*-gentiobiose [20,21], strains APL15 and APL16 were differentiated from *Lc. lactis* subsp. *hordniae* and *Lc. lactis* subsp. *tructae*, and were corresponded to those for *Lc. lactis* subsp. *cremoris* (Table 2). Genotypic and phenotypic test results demonstrated that APL15 and APL16 were identified as *Lc. lactis* subsp. *cremoris* (recently reclassified as *Lc. cremoris* subsp. *cremoris* [22]).

#### EPS production

**Figure 3:**
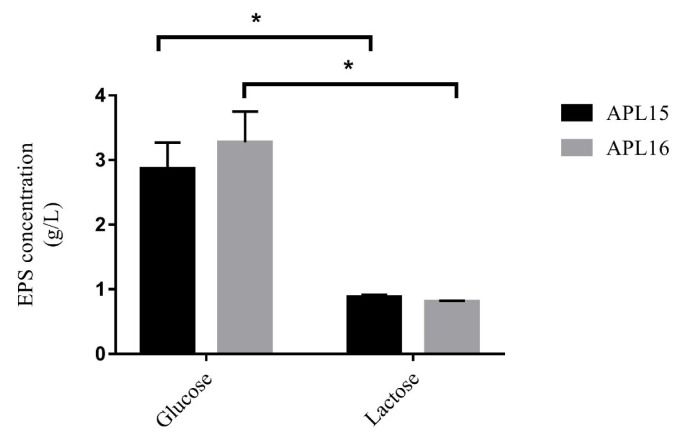
Exopolysaccharide production of APL15 and APL16 in modified MRS broth after cultivation at 26°C for 24 h. Glucose and lactose were added at 2% individually as carbon sources. Data are shown as mean±standard deviation (n = 3). * Denotes a significant difference between the two groups (p<0.05).

During the screening process, EPS-like pellets in the cultures of APL15 and APL16 were observed. The EPS released by APL15 and APL16 were both significantly higher in the medium of 2% glucose (2.9 to 3.3 g/L) than those in 2% lactose (0.8 to 0.9 g/L) after cultured for 24 h (Figure 3). Biosynthesis of EPS from lactose is lower than glucose because the former requires sugar degradation of disaccharide into glucose-6P first, before it can be catabolized for the production of sugar biomass [23]. Figure 3 showed that the EPS generated from our strains in glucose and lactose media were 3.1 to 3.5 and 3.0 to 4.0 times higher, respectively, when compared with those of the *Lc. lactis* subsp. *cremoris* strains NIZO B40, NA4010, and LC330 [24,25]. This indicates that APL15 and APL16 might provide more EPS on the making of fermented milk with or without added sugar. Since there was no significant difference in the EPS concentration between the two strains cultured in lactose medium, both APL15 and APL16 were selected for the production of fermented milk.

### Low-fat fermented milk of APL15 and APL16

#### Fermentation characteristics

Strains APL15 and APL16 were activated to evaluate their fermentation abilities in low-fat milk at 26°C. After 16 to 20 h, the pH values of fermented milks FM-APL15 (by strain APL15) and FM-APL16 (by strain APL16) were 4.42±0.05 and 4.46±0.04, respectively (data not shown). Acidifications of APL15 and APL16 in low-fat milk were ended after 24 h with stabilized pH values of 4.25 to 4.29 and titratable acidities of 0.81% (Table 3), as required by the Food and Agriculture Organization of the United Nations (FAO) [26]. Table 3 also showed that the pH values and viable counts of FM-APL15 and FM-APL16 were not significantly different from those of the control low-fat yogurt made from commercial starter cultures (*S. thermophilus* and *Lb. delbrueckii* subsp. *bulgaricus*), whereas the titratable acidities of FM-APL15 and FM-APL16 were significantly higher (p<0.05) than that of the control.

Whey separation is an undesirable effect of syneresis in fermented dairy products as the properties of milk proteins are modified during acidification [27], and it would negatively affect the perception of consumers towards product satisfaction. The degree of syneresis might be influenced by the solid milk content, milk heat treatment, incubation temperature, rate of acidification, and cooling process, as inappropriate processing could alter the gel structure and serum entrapment. The synereses of FM-APL15 and FM-APL16 (11.71% to 12.22%) were significantly lower (p<0.05) than in the control yogurt (39.61%) (Table 3). There is evidence to show that the interactions of EPS strands with milk proteins ameliorated the spontaneous whey separation of low-fat fermented milk observed by cryo-scanning electron microscope [7]. In addition, EPS released by certain LAB provide higher viscosity and sufficient consistency to prevent syneresis and minimize physical damage in final fermented products. Apparently, the viscous property is of primary influence on the quality and stabilization of fermented milk. The viscosities of FM-APL15 and FM-APL16 were 1,069.8 and 1,249.4 mPa·s, respectively, and both values were significantly higher (p<0.05) than that of control yogurt (504.93 mPa·s) (Table 3). The viscosity of FM-APL16 was significantly higher than that of FM-APL15. The viscosity data indicated that EPSs produced from APL15 and APL16 could play an important role in improving viscosity and reduce syneresis in low-fat fermented milk. Although both FM-APL15 and FM-APL16 had better performance in viscosity and stability compared with control yogurt, no significant difference in the EPS production among the three groups were found (p>0.05). The amount of EPS ranged from 160.83 to 177.99 μg/mL (Table 3). Enhancing viscosity might be due to the function of EPS molecules on the configuration of casein micelles and binding with hydration water with no disturbance on the pH value of gelation [28]. All of these implied that APL15 and APL16 improved the physical texture of low-fat fermented milk along with suitable chemical properties.

#### Sensory evaluation

Sensory attributes of FM-APL15 and FM-APL16 were accessed by 30 volunteers with the 9-level hedonic test in five attributes: appearance, aroma, texture, flavor, and overall acceptance (Table 4). The appearance and texture of our products were close to the control low-fat yogurt. Microbial activities of both strains APL15 and APL16 gave good qualities to the rheological properties of low-fat fermented milk without adding food stabilizers. However, the aroma and flavor of our products were lower than the control group, especially FM-APL16 (p<0.05). When the concentration of fat molecules in milk is reduced, amino acids contribute more to the production of flavor compounds. During transamination of aromatic amino acids, aminotransferase from *Lc. lactis* subsp. *cremoris* is different from most other bacteria, as it acts on ketoacids and α-ketoglutarate instead of aspartate and oxaloacetate [29]. This enzymatic system provides a unique taste that is very different from yogurt, which is an important feature in launching a novel product.

#### Storage stability

Storage tests at 4°C for 21 days were performed to assess the stability of fermented samples. During the 0, 7, 14, and 21 days of the storage period (Table 5), the pH value (4.09 to 4.18) and titratable acidity (0.74% to 0.78%) of FM-APL15 and FM-APL16 did not change significantly (p>0.05). Prasanna et al [8] reported, however, that significant pH reductions were observed during the storage of their low-fat fermented milks due to the post-acidification of the microorganisms. On day 14 and 21, the viable cell counts of FM-APL15 (6.67 to 6.27 log CFU/g) were higher than the recommended value of 6 log CFU/g [25], which were also higher (p<0.05) than those in FM-APL16 (6.27 to 4.71 log CFU/g) (Table 5). The survival of probiotic strains in fermented dairy products has been shown to be strain-dependent, and closely related to the types of yogurt starter cultures, species interactions, and acidity. Vinderola et al [30] found that the viability of probiotic strain in yogurt could be facilitated by co-cultivation with yogurt starter cultures: *Lb. delbrueckii* subsp. *bulgaricus* and *S. thermophiles*. Although the viable total counts of FM-APL15 and FM-APL16 decreased during storage for 21 days at 4°C, severe synereses were not observed during storage (p>0.05). This indicated that strains of APL15 and APL16 had great abilities to maintain the textural properties of fermented products after cooling regardless of cell counts. In brief, APL15 offered stable physicochemical parameters to low-fat fermented milk during the 21-day storage.

In conclusion, we have demonstrated that EPS-producing *Lc. lactis* subsp. *cremoris* strain APL15 isolated from Taiwanese ropy fermented milk had good fermentation abilities when applied in the production of low-fat fermented milk. It provided good appearance and sticky character for low-fat fermented milk, and showed no whey separation with sufficient viable cells during the storage period. These data indicate that exopolysaccharides-producing LAB have great potential as natural food stabilizers for low-fat fermented milk.

## Figures and Tables

**Table 1:** BLAST analysis of 16S rRNA gene sequences for APL15 and APL16

Strain	Length (bp)	Species	Accession No.	Identity (%)
APL15	1,514	*Lactococcus lactis* subsp. *cremoris*	NR_040954	100
		*Lactococcus lactis* subsp. *cremoris*	NR_113925	100
		*Lactococcus lactis* subsp. *tructae*	NR_116443	99
APL16	1,517	*Lactococcus lactis* subsp. *cremoris*	NR_040954	99
		*Lactococcus lactis* subsp. *cremoris*	NR_113925	99
		*Lactococcus lactis* subsp. *tructae*	NR_116443	99

**Table 2:** Differential carbohydrate fermentation reactions of strains APL15 and APL16, and their phylogenetically closest neighbors

Carbohydrate	Strains^1)^					

	1	2	3	4	5	6
Amygdalin	−	−	−	V	−	+
Galactose	+	+	+	+	−	+
Lactose	+	+	+	V	−	+
Maltose	−	−	−	+	−	+
Mannitol	−	−	−	V	−	+
Melibiose	−	−	V	−	−	+
Raffinose	−	−	V	−	−	+
Ribose	−	−	−	+	−	+
Sucrose	−	−	−	V	+	+
D-Xylose	−	−	−	+	−	−
*β*-Gentiobiose	−	−	−	+	−	+

Data for strains APL15 and APL16 were obtained in the present study.

Data for the reference strains from Perez et al [20] and Meucci et al [21].

+, positive; −, negative; V, variable.

1) 1, APL15; 2, APL16; 3, *Lactococcus lactis* subsp. *cremoris* (DSM 20069^T^ and LMG 8505); 4, *Lc. lactis* subsp. *lactis* (DSM 20481^T^ and LMG 7930); 5, *Lc. lactis* subsp. *hordniae* (DSM 20450^T^ and LMG 9462); 6, *Lc. lactis* subsp. *tructae* (DSM 21502^T^).

**Table 3:** Physicochemical and phenotypic characteristics of low-fat fermented milk

Items	Control^1)^	FM-APL15^1)^	FM-APL16^1)^
pH value	4.34±0.03	4.29±0.04	4.25±0.05
Titratable acidity (%)	0.72±0.02^a^	0.81±0.00^b^	0.81±0.01^b^
Viable total count (log CFU/g)	9.21±0.10	9.28±0.05	9.20±0.12
Syneresis (%)	39.61±1.12^a^	11.71±0.73^b^	12.22±0.53^b^
Viscosity (mPa·s)	504.93±51.52^a^	1,069.80±38.60^b^	1,249.40±52.57^c^
EPS production (μg/mL)	177.99±22.84	160.83±21.99	170.45±28.49

Data are shown as mean±standard deviation (n = 3).

CFU, colony-forming units; EPS, exopolysaccharides.

1) Control, low-fat yogurt prepared by commercial starter cultures (*Streptococcus thermophilus* and *Lactobacillus delbrueckii* subsp. *bulgaricus*); FM-APL15 and FM-APL16, low-fat milks inoculated with 1% (v/v) *Lactococcus lactis* subsp. *cremoris* APL15 and *Lc. lactis* subsp. *cremoris* APL16 respectively, after fermentation at 26°C for 24 h.

a–c Within a row, different superscript letters denote significant differences between the groups (p<0.05).

**Table 4:** Sensory evaluation by consumer acceptance test

Items	Control^1)^	FM-APL15^1)^	FM-APL16^1)^
Appearance	7.27±1.62^a^	7.10±1.30^a^	6.80±1.40^a^
Aroma	7.57±1.48^a^	6.73±1.36^ab^	6.53±1.43^b^
Texture	7.00±1.91^a^	6.87±1.48^a^	6.23±1.77^a^
Flavor	7.47±1.48^a^	6.53±1.83^ab^	6.27±1.78^b^
Overall	7.63±1.52^a^	6.80±1.47^ab^	6.60±1.50^b^

Data are shown as mean±standard deviation (n = 30).

1) Control, commercial low-fat yogurt; FM-APL15 and FM-APL16, low-fat milks inoculated with 1% (v/v) *Lactococcus lactis* subsp. *cremoris* APL15 and *Lc. lactis* subsp. *cremoris* APL16 respectively, after fermentation at 26°C for 24 h.

a,b Within a row, different superscript letters denote significant differences between the groups (p<0.05).

**Table 5:** Storage conditions of fermented milks at 4°C for 21 days

Items	Storage period (d)			

	1	7	14	21
pH				
FM-APL15^1)^	4.18±0.03	4.12±0.02	4.16±0.04	4.12±0.02
FM-APL16^1)^	4.18±0.02	4.09±0.01	4.12±0.00	4.11±0.01
Titratable acidity (%)				
FM-APL15	0.76±0.01	0.74±0.04	0.77±0.01	0.77±0.01
FM-APL16	0.76±0.01	0.74±0.03	0.78±0.01	0.77±0.01
Viable count (log CFU/mL)				
FM-APL15	8.82±0.38^a^	8.87±0.14^a^	6.67±0.05^b^,^X^	6.27±0.42^b^,^X^
FM-APL16	8.75±0.07^a^	8.59±0.19^a^	6.30±0.14^b^,^Y^	4.71±0.58^c^,^Y^
Syneresis (%)				
FM-APL15	4.83±2.16	8.77±2.91	8.12±1.25^X^	6.70±1.52
FM-APL16	5.12±0.72	7.43±1.00	5.60±0.07^Y^	4.73±2.05

Data are shown as mean±standard deviation (n = 3).

CFU, colony-forming units.

1) FM-APL15 and FM-APL16, low-fat milks inoculated with 1% (v/v) *Lactococcus lactis* subsp. *cremoris* APL15 and *Lc. lactis* subsp. *cremoris* APL16 respectively, after fermentation at 26°C for 24 h.

a–c Within a row, different superscript letters denote significant differences between the groups (p<0.05).

X,Y Within a column, different superscript letters denote significant differences between the groups (p<0.05).

## SUPPLEMENTARY MATERIAL

**Supplementary Figure S1.** Partial sequences of the 16S rRNA gene of the strains APL15 and APL16 to their related species in the genus Lactococcus.
