# Supplementary material for: Characterization of exopolysaccharide-producing lactic acid bacteria from Taiwanese ropy fermented milk and their application in low-fat fermented milk
Source: Anim Biosci. 2021 Aug 25;35(2):281–9. doi: 10.5713/ab.21.0251 (PMC8738951; doi:10.5713/ab.21.0251)
Supplement: Supplementary file 1 [file ab-21-0251-suppl.pdf]

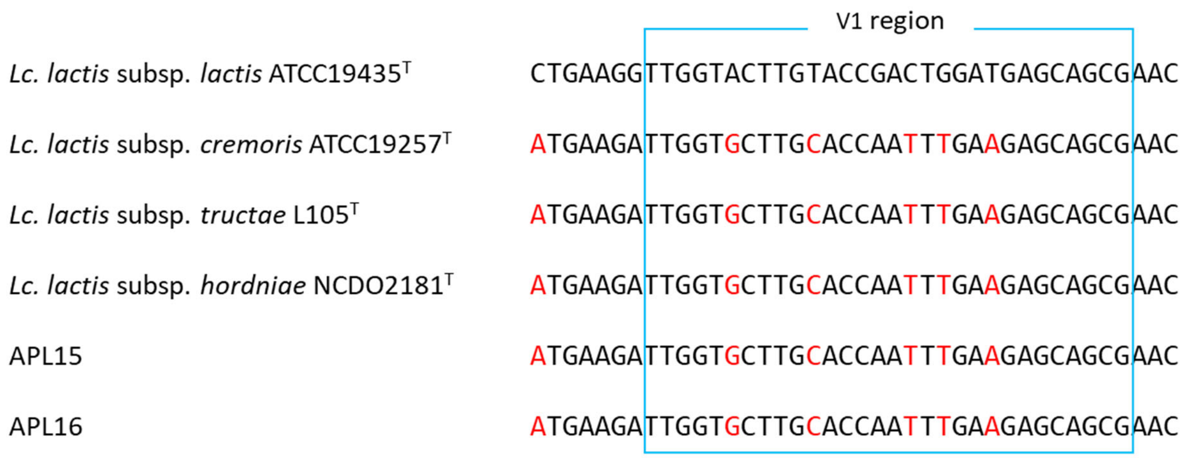

Supplementary Figure S1. Partial sequences of the 16S rRNA gene of the strains APL15 and APL16 to their related species in the genus *Lactococcus*. V1 region is positioned from 79 to 110 with respect to *Escherichia coli* 16S rRNA gene numbering. Bases different from those of *Lc. lactis* subsp. *lactis* are indicated in red.
